# Soil fertility and plant nutrition in an organic olive orchard after 5 years of amendment with compost, biochar or their blend

**DOI:** 10.1038/s41598-024-67565-x

**Published:** 2024-07-18

**Authors:** Fernando Fornes, Antonio Lidón, Rosa M. Belda, Giovana P. F. Macan, María L. Cayuela, María Sánchez-García, Miguel A. Sánchez-Monedero

**Affiliations:** 1 Departamento de Producción Vegetal, Universitat Politècnica de València, 46022 Valencia, Spain; 2 Instituto Universitario de Ingeniería del Agua y del Medio Ambiente, Universitat Politècnica de València, 46022 Valencia, Spain; 3 Department of Soil and Water Conservation and Organic Waste Management, CEBAS-CSIC, 30100 Murcia, Spain

**Keywords:** Biochar:compost blend, Olive plantlet, Soil microbial activity, Soil nutritional status, Soil physical properties, Carbon cycle, Agroecology

## Abstract

The agronomic use of compost and biochar as soil amendments may exhibit contrasting results in terms of soil fertility and plant nutrition. The effects of the biennial application of biochar, compost and a blend of compost:biochar (90:10; % dw:dw) on the agronomical performance of an organically managed and well established 25-year-old olive orchard was assessed 5 years after the initial application. The agronomical evaluation was based on the assessment of the soil physical, chemical, and biological characteristics, and the assessment of the soil fertility by both crop production and nutritional status of the orchard, and the bioassay with olive plantlets. Biochar mainly benefited the physical properties (bulk density, total porosity, aeration, water retention capacity) of soil, especially in the top 0–5 cm. Compost and its blend with biochar improved microbial activity, soil nutritional status (increasing the content of soluble organic C, N, and P) and favoured the formation of aggregates in soil. The bioassay conducted with young plantlets confirmed the enhanced soil fertility status in the three amended treatments, particularly in the case of biochar and its blend with compost. However, this effect was not significantly observed in the adult plants after 5 years of application, reflecting the slow response of adult olive trees to changes in fertilization. Based on these results, alongside the desirable long-residence time of biochar in soil and the ready availability of compost, the blend of biochar with compost assayed in this study is defined as a valid strategy for preparing high quality soil organic amendments.

## Introduction

The olive tree is an ancient plant culture in the Mediterranean basin that represents an important cultural and economic background in the area. Olive trees are adapted to dry conditions, although an adequate supply of water is needed in order to obtain high yields. They are also well adapted to extreme temperatures, i.e. hot in the summer and cold in the winter^1^. Soils in the Mediterranean olive groves are usually calcareous and poor in organic matter, requiring the regular application of organic amendments^2^. Moreover, tree crop orchards often show soil physical and hydrological deterioration due to several factors related with the limited subsoiling agrotechniques that can be implemented during the long life cycle of these crops^3^. In this context, the application of organic amendments into the soil is a good strategy to alleviate soil compaction and restore not only the physical properties, but also to improve the chemical and biological status of the soil^4^. Traditionally, green and animal manures, preferentially composted, have been added to these soils^5^, although more recently, other types of organic wastes (i.e. sewage sludge, digestates) have been applied as soil amendments^6,7^. The olive agro-industry produce organic wastes such as olive tree pruning, olive stones, and two-phase olive mill wastes, which are susceptible to be recycled as organic amendments^8–10^. Composting and pyrolysis are two alternative methods to treat, stabilize and sanitize organic wastes, allowing their safe use as soil amendments or as soilless growth media constituents.

Composting is the controlled aerobic decomposition of organic matter carried out by different populations of microorganisms. It is a reliable and widely spread process for the treatment of urban and farming organic wastes. The characteristics of the compost could be highly variable, mainly depending on the raw material and the composting process^11^. High quality compost must be a stabilized, nutrient rich material that is useful as organic-mineral fertilizer, and it has been traditionally recognized as an excellent material to be applied in tree crop cultivation^12,13^. Compost not only supplies minerals and humic-like substances to the soil, but it can also enhance soil microbial diversity and activity^14,15^. Compost can modify some physicochemical characteristics of the soil, such as pH^16,17^ and cation exchange capacity (CEC)^18^, and can also improve the soil’s physical properties by favouring the formation of aggregates^19^, and hence the porosity and soil water retention^20^.

More recently, biochar, obtained by pyrolysis of biomass, has emerged as an alternative soil organic amendment. Biochar is a carbonaceous organic material that is usually porous, alkaline, variable in available nutrient content, and recalcitrant to decomposition^21,22^. Fresh biochar, particularly when prepared at high temperatures, has a low CEC in comparison with soil organic matter^23^, although aged or oxidized biochar is rich in functional groups that confer a high CEC^23,24^. Hence, although the biochar’s characteristics are variable and dependent on the original feedstock and the pyrolysis conditions (i.e. processing temperature and residence time, use of additives, etc.)^25^, it is a properly stabilized and sanitized organic material ready to be used as organic amendment for agricultural soils, especially when it comes to poor and/or degraded soils^26,27^. Biochar improves the soil’s physical properties by decreasing bulk density and particle density, increasing porosity, improving wet aggregate stability, and increasing water retention and water availability to plants, by balancing the water infiltration and the saturated and unsaturated hydraulic conductivity^28^.

Compost typically outperforms biochar in providing a larger supply of nutrients and microbiota^10^, whilst biochar outperforms compost in enhancing levels of soil organic matter because of its large supply of recalcitrant carbon^29^, and its ability to reduce greenhouse gas emissions^30^. The decision regarding whether to use compost or biochar as a soil amendment should consider other factors beyond the agronomical performance, such as its availability, price, environmental and health concerns, etc. In this sense, compost is easily available at a low price whilst biochar is expensive and not widely available^9^. Accordingly, it could be interesting to explore the combination of compost with biochar, where compost is the main constituent in the blend.

The literature available supports the hypothesis that a blend of compost and biochar will have a synergistic effect for improving the properties and agronomical performance over both materials separately^26,31^. Positive results have been obtained with blends of compost with biochar blended before composting^32^ or after composting^33^. However, more studies are needed on the long-term effects of compost, biochar, or compost-biochar blends on orchard soils^26^.

Our study is based on the work initiated by Sánchez-García et al.^10^. These authors studied the short-term effect, for two years, of soil amendment with compost, biochar, and a combination of both materials, on carbon and nitrogen dynamics in an adult olive orchard in South-Eastern Spain. In that study, the effects of the amendment on the physical properties of the soil were not investigated, and it was not possible to detect any significant effect on plant growth in adult trees due to their slow response to fertilization.

The main objective of the present study was to analyse the medium-term effects of biochar, compost, and their blend, after 5 years of recurrent application, on the soil’s physical and biological properties of an adult olive orchard. A second objective was to assess the effect of the same treatments on soil fertility, by evaluating crop production and nutrient status of the orchard, and through a bioassay with olive plantlets grown in pots. This second objective anticipates the slow response of well-established trees compared to the bioassay experiment. With this, we intent to confirm the hypothesis that amending the soil with a blend of compost with biochar outperforms treatments where compost or biochar are added alone. The findings of this medium-term assessment are expected to further guide the selection of the most effective fertilization practices involving the use of biochar and compost in olive orchards.

## Material and methods

### Description of the field scale experiment.

The experiment was conducted in a 25-year-old commercial organic olive grove in the farm “SAT Casa Pareja”, Jumilla, Spain (38°23′N; 1°22′W). The soil was a Haplic Calcisol^34^, composed of 57% sand and 16% clay, 30% carbonate, and 1.68% total organic C (TOC). In 2013, an experimental field was established in the olive orchard^10^. The experiment consisted of the following four treatments, with three replicates each: (i) no amendment (control), (ii) compost, (iii) a blend of compost:biochar at 90:10 ratio (dw:dw) and (iv) biochar. The amendments were applied in 2013, 2015, and 2017. Biochar was produced by a local provider from holm oak wood by slow pyrolysis at 650°. This biochar is characterised by a rich organic C composition with a high degree of aromaticity. Similar batches of biochar, ground to less of 10 mm were used for the three applications. Composts were prepared every year in the farm mixing two-phase olive mill waste with sheep manure and olive tree pruning in windrows with regular temperature monitoring and moisture control over a six-month period^35^. The average composition of the amendments used during the five-year experiment is shown in Table 1^10,35,36^. The nature and characteristics of those amendments, and how they were applied, have been described by Sánchez-García et al.^10^ and reproduced here in Table 1.

**Table 1:** Range of chemical composition of the organic amendments used during the three applications (results expressed in dry weight).

Parameter (unit)	Biochar	Compost
pH^a^	9.26—10.30	8.55—8.71
EC^a^ (dS m^−1^)	0.57—0.60	2.70—7.17
TOC (%)	67.3—76.5	32.6—35.8
TN (%)	0.80—0.84	2.35—2.40
P (%)	0.19—0.20	0.38—1.1
K (%)	0.60 – 0.72	1.7—3.6
DOC^a^ (g kg^−1^)	0.38	32.1
NH_4_^+^-N^b^ (mg kg^−1^)	3.20	120—189
NO_3_^-^ -N^a^ (mg kg^−1^)	0.8	118—140
BET surface area, N_2_ (m^2^ g^−1^)	280.0	–

^a^Water extract; ^b^KCl 2 M solution extract; BET: Brunauer, Emmett and Teller.

Data obtained from Sánchez-García et al., ^10^, López-Cano et al., ^35^ and Takaya et al., ^36^.

### Soil sampling and characterization

#### Soil physical properties

The physical properties of the soil, such as bulk density (BD), total porosity (TP), soil water retention curve (SWRC), pore distribution (PD), saturated hydraulic conductivity (Ksat), hydraulic conductivity near saturation (K(h)), and aggregation percentage (AP) were studied. SWRC and PD were determined in undisturbed soil samples, collected using an auger with two soil sample rings 5.3 cm in diameter and 3.0 cm in height (S1). Six soil samples were taken in each treatment, three at a depth of 0–5 cm and three at a depth of 5–10 cm. BD, TP, Ksat, K(h) and AP were determined in undisturbed soil core samples, collected with an auger containing a single ring measuring 7.6 cm in diameter and 7.6 cm in height (S2). For this purpose, three soil samples per treatment were taken in the 0–10 cm depth interval.

All soil samples collected with the small rings (0–5 cm and 5–10 cm) (S1) were slowly fully saturated by placing soil cores on the top of a porous brick soaked in water to avoid trapped air bubbles that could alter the soil structure. The soil samples were kept in the bricks until they were saturated and then transferred to a pressure plate apparatus (Soil moisture Equipment Corp., USA) to establish a wet to dry sequence with six soil-matric potentials (− 10, − 20, − 30, − 60, − 90 and − 300 kPa) for the construction of the SWRC^37^. The pairs of volumetric moisture and matric potential data allowed us to fit Campbell’s equation^38^, and thus to estimate the volumetric water content at the permanent wilting point (− 1500 kPa) and in the range near saturation (0 to − 10 kPa). The water content at each potential was used to estimate the pore distribution according to Kay^39^. The mean pore diameter (MPD) was also determined as the weighted sum of the relative water content remaining in the soil at each pressure applied, multiplied by the mean pore diameter of the considered interval.

Undisturbed soil cores obtained with the big rings (S2) were placed on a porous brick with water so that all pores were filled by capillary action without affecting the soil structure. A laboratory constant-head permeameter was used to determine K_sat_ (cm h^-1^), by measuring the difference in water levels and collecting the drained water in a burette during a fixed period of time, when the water was flowing constantly through an undisturbed and saturated soil sample. When also knowing the difference in hydraulic head, the distance over which it is applied, and the sample cross-section area, it is possible to calculate the K_sat_ according to Darcy’s law^40^. Once the hydraulic conductivity was determined, the samples were air-dried and the near-saturated hydraulic conductivity was obtained from mini disk infiltrometer data (METER Group, Inc., Pullman, WA, USA). Four consecutive suction rates (− 0.5, − 2, − 4 and − 6 cm) were applied to each soil sample, and the water volume was recorded at regular time intervals as the water infiltrated. The measured infiltration data were analysed based on the steady-state data analysis method proposed by Zhang^41^ which calculates the K(h) (cm h^−1^).

Subsequently, the BD (g cm^−3^) was determined with the gravimetric method, based on the ratio of the mass of soil dried at 105 °C to the soil sample volume. TP (cm^3^ cm^−3^) was calculated based on soil particle density (PD), assuming a value of 2.65 g cm^−3^, and bulk density. Finally, these samples were used to determine the aggregate-size distribution. For this purpose, the soil was fractionated into aggregates by a dry-sieving method, according to the methodology described by Gartzia-Bengoetxea et al.^42^. Each soil sample was placed above nested sieves mounted on a Microcomputer Screener FT-91 sieve shaker (Filtra Vibrations S.L., Barcelona, Spain). The sieves were mechanically shaken at medium speed for 2 min to separate soil into the following aggregate-size classes: > 25, 25–10, 10–4, 4–2 mm (large macroaggregates), 2.0–0.25 mm (macroaggregates), 0.25–0.053 mm (microaggregates), and < 0.053 mm (silt and clay size fraction). Then, the mean weight diameter (MWD) of each sample was determined as the sum of the product of the percentage of sample weighted on the sieve by the mean diameter of the size classes.

#### Soil physicochemical, chemical and biochemical properties

To study the soil’s physicochemical, chemical and biological properties in the laboratory, soil samples (three replicates per treatment) were obtained by digging on the 15 cm surface layer of the soil. The samples were immediately transported to the laboratory and sieved through a 2 mm mesh. They were stored in parafilm sealed bottles at 4 ℃ in the dark.

The main physicochemical characteristics, pH, and EC, as well as the available mineral content of the soils, were determined in a 1:5 (w:v) soil:water suspension as indicated in the European Standards (EN 13037 1999; EN 13038 1999; EN 13652 2001, respectively) for soil improvers and growing media. The exception was NH_4_^+^-N which was extracted with KCl 2 M (1:5, w:v). The pH was measured using a Crison model 2000 pH meter. EC was determined with a Crison model 522 conductimeter. Dissolved organic C (DOC) and dissolved N (DN) were determined using a Photometer (Nanocolor 500 D MACHEREY–NAGEL, Germany). DOC was measured after removing all inorganic carbon. NH_4_^+^ was determined by a colorimetric method based on Berthelot’s reaction^43^. NO_3_^−^ was determined by ion chromatography (HPLC, model 861, Metrohm AG, Herisau, Switzerland), and P, K, Ca, and Mg were determined using reflectoquant technology (Merck^®^; Darmstadt, Germany): The analyses were performed with a reflectometer RQflex 10 Reflectoquant, using the corresponding barcode strips for calibration and test strips for nutrient quantification, following the manufacturer’s instructions. Dissolved organic N (DON) was calculated by the difference between DN and the mineral N forms (NO_3_^-^-N, NH_4_^+^-N).

The Bradford-reactive soil protein N (BRSP-N) was analysed following the method proposed by Wright et al.^44^, as described by Gómez-Bellot et al.^45^. Briefly, one gram of dry 2 mm sieved soil was blended with 8 mL of 50 mM sodium citrate, pH 8.0, and extracted by autoclaving at 121 °C for 90 min, and centrifuging at 5000 rpm for 15 min. The pellet was re-extracted several times until a clear colourless extract was obtained. The protein in the supernatant was determined by the Bradford dye-binding assay with bovine serum albumin as the standard. The BRSP-N was calculated assuming that proteins contain 16% N^46^.

All these determinations were performed in triplicate.

The microbial activity was monitored on fresh soil samples in the laboratory by i) soil respiration, and ii) the capacity to hydrolyse fluorescein diacetate (FDA). Soil respiration was measured as the CO_2_ emitted by the soil samples using a CheckPoint portable gas analyser (Dansensor, Ringsted, Denmark). The rate of FDA hydrolysis was determined by the method proposed by Schnürer and Rosswall^47^.

### Plant nutritional assessment

#### Crop yield and nutritional status of the olive trees

Olive crop yield was estimated in January 2018 by harvesting and weighing the combined olive production of the six trees in each plot. Crop yield was expressed as tons of olives per hectare. The nutritional status of the olive trees was assessed by foliar analyses performed on leaf samples according to Fernández-Escobar et al.^48^. Twelve leaves from one-year-old shoots, located in basal to middle positions, were sampled from each tree (total: 216 leaves per treatment) in July. Leaves were washed with deionised water, oven dried at 60 °C for 72 h and milled for the analysis of N, P, K, Ca, and Mg. N was determined by elemental analysis (Flash EA 1112 Series-LECO Truspec). P, K, Ca, and Mg were determined by Inductively Coupled Plasma Atomic Emission Spectrophotometry (ICP-AES; Agilent 5110).

#### Plant growth bioassay

A plant growth bioassay was performed with uniform rooted cuttings of olive cv. ‘Arbequina’, at the four- to six-leaf developmental stage, obtained from Hernandorena, a commercial nursery located in Benimodo (Spain). The rooted cuttings were planted in 1 L pots (1 cutting per pot) containing the studied soils. Twelve pots per treatment were randomly distributed following a block design of 4 blocks with 3 pots each, in a climatic greenhouse equipped with heating and cooling systems. The soils were fertilized with 5 g of a controlled release fertilizer (Osmocote ^®^Plus, 6 months) per pot. Plants were watered as needed with micro-sprinklers, and grown for 10 months. At the end of the experiment, relevant plant growth parameters (stem fresh weight, number of leaves, and root fresh weight) were recorded. Dry leaf tissue was finely ground for analysis of N, P, K, Ca, and Mg, as described above.

### Data analysis

The results were statistically evaluated with an analysis of variance (ANOVA). When significant differences were found in the ANOVAs, Tukey tests at P ≤ 0.05 were carried out. In the case of soil physical properties, a significance level of P < 0.10 was adopted considering the spatial variability observed in field-scale experiments.

Statistical analyses were performed with the Statgraphics Centurion XVII statistical package (2020 Statgraphics Technologies, Inc., The Plains, Virginia, USA).

## Results

### Soil physical properties

**Figure 1:**
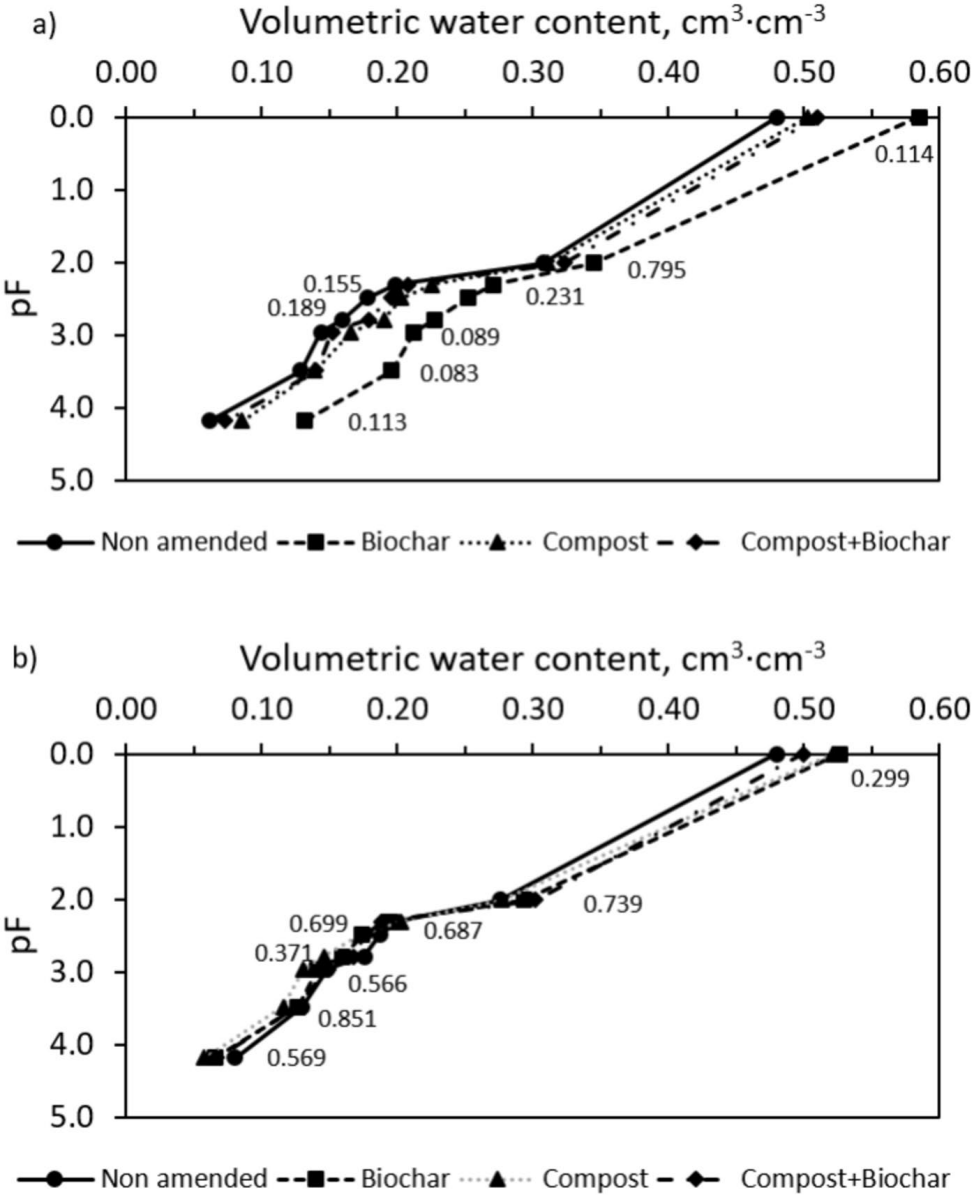
Soil water retention curves for each treatment in a) the 0–5 cm layer and b) in the 5–10 cm layer. Matric head (h) expressed as pF value (–log h, with h in cm). Figures refer to P-values for each matric head/soil moisture pair.

Table 2 shows the selected soil physical properties of the different treatments after 5 years of biennial application. In general, these properties were highly variable. However, despite the low statistical significance, there were some interesting differences for the unsaturated hydraulic conductivity (P = 0.10), bulk density (P = 0.17), and total porosity (P = 0.17). The biochar treatment showed a stronger effect on soil physical properties, particularly on bulk density and porosity, as compared to compost and the blend of compost and biochar. The impact of biochar on soil physical properties was also reflected in the hydraulic conductivity in near-saturated conditions, shown in Table 2. Whereas data on saturated hydraulic conductivity showed a large variability, the hydraulic conductivity measured at different tensions showed a decreasing trend due to the addition of the different amendments. From all treatments, biochar showed the lowest conductivity values as compared to unamended soils under -0.5 tension (P = 0.10). Biochar also increased the soil water retention capacity at low and high, but not intermediate, matric potentials (Fig. 1). This effect was more pronounced in the topsoil (0–5 cm) (Fig. 1a) than in the lower soil layer (5–10 cm) (Fig. 1b). The volumetric water content at field capacity (− 33 kPa) ranged from 0.1776 to 0.2517 cm^3^ cm^−3^ in the topsoil, with the highest value corresponding to the soil treated with biochar and the lowest to the untreated soil. Although water content at the permanent wilting point (− 1500 kPa) was similar in all the treatments, the available water for plants showed an increasing trend, between 2.9 and 4.4% as compared to control soil. A higher water retention capacity of the biochar-treated soil was observed, which also showed an increasing trend in microporosity as compared to the unamended soil.

**Table 2:** Effects of soil amendment on physical properties such as bulk density (BD, g cm^−3^), total porosity (TP, %v/v), saturated hydraulic conductivity (K_SAT_; mm d^−1^), unsaturated hydraulic conductivity at different applied tensions (K_0.5_, K_2_, K_4_ and K_6_ for − 0.005 m, − 0.02 m, − 0.04 m and − 0.06 m, respectively; mm d^−1^), percentage of macroaggregates (2.0–0.25 mm, MAP, %), percentage of microaggregates (0.25–0.053 mm, mAP, %) and the mean weight diameter of aggregates (MWD, mm).

Treatment		BD	TP	K_SAT_	K_0.5_	K_2_	K_4_	K_6_	MAP	mAP	MWD
Non amended	Mean	1.426	46.2	1637	967	799	641	368	19.5	37.5	0.376
	*SD*	*0.104*	*3.9*	*283*	*174*	*243*	*98*	*203*	*1.8*	*8.1*	*0.066*
Biochar	Mean	1.292	51.2	1562	614	614	401	418	21.7	41.8	0.466
	*SD*	*0.091*	*3.4*	*293*	*155*	*184*	*155*	*269*	*2.7*	*4.3*	*0.071*
Compost	Mean	1.316	50.4	1556	785	577	446	277	23.5	38.9	0.538
	*SD*	*0.102*	*3.8*	*570*	*175*	*173*	*209*	*68*	*3.5*	*3.2*	*0.191*
Compost + Biochar	Mean	1.389	47.6	1288	865	719	574	513	20.2	42.3	0.438
	*SD*	*0.053*	*2.0*	*188*	*91*	*133*	*2*	*213*	*3.1*	*2.9*	*0.118*
P-value		0.176	0.177	0.671	0.104	0.496	0.205	0.772	0.255	0.509	0.427

*SD* standard deviation from the mean (n = 3).

All treatments tended to increase the aggregate fractions obtained by dry-sieving with respect to the unamended soil, although no significant differences were obtained (Table 2). The variation in the percentage of stable aggregates was in line with an increase in the mean weight diameter of aggregates, especially in the case of compost addition.

### Soil physicochemical, chemical and biological characteristics

Table 3 shows some relevant physicochemical and chemical characteristics of the soils. All soils were markedly alkaline, and the compost, alone or with biochar, caused the highest increase in pH. EC was low and within a similar range in all cases. Compost, alone or blended with biochar, markedly increased the concentration of DOC and dissolved nutrients, particularly organic N (DON), P, and K. The extractable fraction of soil protein-N (BRSP-N) also significantly increased by the addition of compost and the compost and biochar blend. Compost, mainly when blended with biochar, but not biochar alone, increased the amount of NO_3_^-^-N. The content of NH_4_^+^-N was negligible in all treatments. Mg increased due to biochar amendment whilst both Ca and Mg decreased in compost and compost-biochar treated soils.

**Table 3:** Effect of soil amendment on the pH, electrical conductivity (EC), dissolved organic carbon (DOC), Bradford-reactive soil protein nitrogen (BRSP-N), dissolved nitrogen (DN), dissolved organic nitrogen (DON), and water-soluble mineral nutrients.

Soil amendment		pH	EC (dS m^-1^)	DOC (mg kg^-1^)	BRSP-N (mg kg^-1^)	DN (mg kg^-1^)	DON (mg kg^-1^)	NH_4_^+^-N (mg kg^-1^)^z^	NO_3_^-^-N (mg kg^-1^)	PO_4_^3-^-P (mg kg^-1^)	K (mg kg^-1^)	Ca (mg kg^-1^)	Mg (mg kg^-1^)
Non amended	Mean	8.6c	0.30	280c	197b	36.5b	22.2b	0.1	14.2a	8c	64c	139a	42b
	*SD*	*0.05*	*0.05*	*20*	*3*	*1.0*	*0.8*	*0.2*	*1.5*	*3*	*9*	*9*	*8*
Biochar	Mean	8.7bc	0.30	280c	213b	41.9ab	27.1b	< 0.1	14.9ab	13bc	62c	133a	68a
	*SD*	*0.05*	*0.04*	*10*	*15*	*9.3*	*4.6*	*-*	*4.7*	*3*	*9*	*9*	*9*
Compost	Mean	8.9ab	0.32	450a	359a	63.5ab	42.9a	< 0.1	20.5ab	18ab	343a	76b	23c
	*SD*	*0.32*	*0.03*	*10*	*14*	*11.2*	*6.5*		*5.2*	*3*	*26*	*5*	*3*
Compost + Biochar	Mean	9.1a	0.36	380b	341a	66.2a	40.0a	< 0.1	26.2b	24a	293b	66b	22c
	*SD*	*0.10*	*0.04*	*20*	*3*	*17.0*	*12.2*	*-*	*6.0*	*6*	*18*	*4*	*3*
Significance^y^		**	ns	**	***	*	*	ns	*	***	***	***	***

^z^NH_4_^+^-N was determined in KCl 2 M extracts (1:5, w:v).

^y^ns = non-significant; * significant at P ≤ 0.05; ** significant at P ≤ 0.01; ***significant at P ≤ 0.001. *SD* standard deviation from the mean (n = 3).

Compost alone or blended with biochar increased the soil microbial activity measured in terms of both FDA hydrolysing activity and soil microbial respiration (Table 4). The use of biochar alone only caused a slight increase in the FDA hydrolysing activity compared to the control, but markedly lower than the effect of compost application, with no significant impact on soil respiration.

**Table 4:** Effect of soil amendment on soil microbial activity measured as the hydrolysis of fluorescein diacetate (FDA) and as the respiratory emission of CO_2_.

Treatment		FDA hydrolysed (mg kg soil^−1^ day^−1^)	CO_2_ Emitted (mg kg soil^−1^ day^−1^)
Non amended	Mean	74d	47b
	*SD*	*3*	*2*
Biochar	Mean	89c	48b
	*SD*	*1*	*5*
Compost	Mean	103b	68a
	*SD*	*2*	*4*
Compost + Biochar	Mean	116a	70a
	*SD*	*12*	*4*
Significance^z^		***	***

^z^***significant at P ≤ 0.001. *SD* standard deviation from the mean (n = 3).

### Nutritional status and productivity of the olive orchard

The leaf analysis of the 25-year-old trees did not show any effect on the nutritional status of the orchard caused by the application of the amendments (Table 5). The crop yield was likewise unaffected by any of the treatments.

**Table 5:** Crop production and leaf nutrient content in adult olive trees. Data from the field experiment five years after the first amendment.

Treatment		Crop production (t ha^-1^)	Leaf nutrient content (% dry weight)				
			N	P	K	Ca	Mg
Non-amended	Mean	6.96	1.49	0.11	1.44	0.80	0.16
	*SD*	*1.10*	*0.12*	*0.01*	*0.06*	*0.06*	*0.02*
Biochar	Mean	8.71	1.49	0.10	1.44	0.81	0.17
	*SD*	*1.40*	*0.15*	*0.01*	*0.07*	*0.08*	*0.03*
Compost	Mean	7.75	1.54	0.11	1.41	0.77	0.16
	*SD*	*0.66*	*0.16*	*0.01*	*0.04*	*0.15*	*0.03*
Compost + Biochar	Mean	7.44	1.69	0.11	1.43	0.82	0.18
	*SD*	*1.12*	*0.17*	*0.01*	*0.06*	*0.06*	*0.01*
Significance^z^		ns	ns	ns	ns	ns	ns

^z^ns = non-significant. *SD* Standard deviation from the mean (n = 3).

The plant growth bioassay showed that the three amendments enhanced the growth of olive plantlets, compared to the unamended soil (Table 6). Biochar alone or blended with compost gave the best results on both shoot and root growth compared to compost. The concentration of main nutrients in the leaves was also affected by the amendments. Compost and mainly biochar but not their blend decreased the content of nitrogen. Phosphorus was not affected in any of the cases. The concentration of potassium increased in both treatments containing compost, especially in the one blended with biochar. The concentration of calcium and magnesium in leaves decreased with the three amendments.

**Table 6:** Effect of soil amendment on growth and nutrient content in the leaves of plantlets grown in pots.

Treatment		Plant biomass (g fresh weight /plant)			Leaf nutrient content (% dry weight)				
		Shoot	Root	Nº of leaves	N	P	K	Ca	Mg
Non amended	Mean	2.98b	0.65b	23.0b	3.28a	0.14	1.34c	0.90a	0.16a
	*SD*	*0.12*	*0.11*	*1.8*	*0.07*	*0.02*	*0.15*	*0.13*	*0.02*
Biochar	Mean	3.85a	0.94ab	26.5ab	2.61c	0.14	1.37c	0.68b	0.13b
	*SD*	*0.12*	*0.08*	*0.6*	*0.03*	*0.01*	*0.03*	*0.08*	*0.01*
Compost	Mean	3.40ab	0.73b	25.3ab	2.90b	0.13	1.70b	0.64b	0.11bc
	*SD*	*0.12*	*0.11*	*2.6*	*0.03*	*0.02*	*0.05*	*0.13*	*0.02*
Compost + Biochar	Mean	3.45ab	1.45a	27.0a	3.18a	0.13	2.27a	0.62b	0.10c
	*SD*	*0.61*	*0.51*	*1.5*	*0.22*	*0.02*	*0.30*	*0.03*	*0.01*
Significance^z^		*	**	*	***	ns	***	**	***

^z^ns = non-significant; * significant at P ≤ 0.05; ** significant at P ≤ 0.01; ***significant at P ≤ 0.001. *SD* standard deviation from the mean (n = 4).

## Discussion

The objective of this study was to investigate the influence of compost, biochar and their combination, on the soil physical, chemical and biological characteristics of an olive orchard, and to assess soil fertility in terms of crop yield, plant nutritional status, and a plant growth bioassay.

### Impact of the organic amendments on soil characteristics

Despite the large variability in physical properties typically observed in field scale experiments^49^, the results showed a shift towards an improvement of the soil physical properties, particularly in the biochar treatment (Table 2 and Fig. 1).

After three applications of the amendments over five years (2013; 2015; 2017), biochar was the most effective in improving soil physical properties, as it increased porosity (implicitly increasing the aeration) whilst increasing the water retention capacity, especially in the top 5 cm. This increase in soil water retention could be due to a change in pore size distribution, through the increase in micropores, which can hold water more strongly than macropores or mesopores, via capillary and adhesive forces. These changes in total porosity, pore size distribution, water transmission, and water retention characteristics of the soil treated with biochar have been previously reported by Blanco-Canqui^50^, who pointed out that these changes are more evident in coarse textured soils, as in our case. The increase in water holding capacity of soil caused by biochar has been previously reported in sandy soils^32,33^, similar to that used in our study, and also in silty loamy soils^51^.

Biochar is a porous material with a high specific surface area, which provides it with certain capacities to modify the physical and hydraulic properties of the soil when used as a soil amendment, alone or in combination with other organic products. The impact on soil physical properties may depend on the properties of the organic amendments (particle size, bulk density, open or occluded porosity, etc.;^52^. Contrasting findings have been found in the literature regarding the impact of different amendments on soil physical properties. Khorram et al.^53^ found that a blend of compost and biochar increased the water holding capacity of soil in an apple orchard, but not the compost or the biochar alone. D’Hose et al.^16^ reported that soil subjected to an arable cropping system retained more water when amended with compost alone or blended with biochar, than when amended with biochar alone. Douh et al.^54^ observed the same effect of compost and biochar in terms of increasing water retention by a soil from an olive orchard. Moreover, other factors such as surface chemistry and feedstock source, which control the hydrophobicity and porosity of the material, along with the production temperature, are also determinant for the ability of biochar to improve soil water holding capacity^55^. In any case, the biochar’s ability to increase the water retention capacity of soils, as found in our experiment, could be an important tool in the dry Mediterranean climate, where the rainwater input is low and irregular, as it may contribute towards the reduction in the frequency of irrigation.

On the other hand, compost and the compost:biochar blend were the most effective amendments for enhancing soil chemical and biochemical properties after 5 years of application. Compost, alone or blended with biochar, supplied significantly more DOC and nutrients than biochar (Table 1). Likewise, the soil available N, both DON and NO_3_^-^, was larger in the compost and the compost-biochar-blend amended soils than in the other treatments (Table 3). Soil biological activity was mostly affected by the compost amendment (Table 4). Both alone or blended with biochar, compost increased all parameters related with the biological and biochemical activity in soil (i.e. microbial hydrolytic activity and respiration). These effects were expected, as compost usually supplies a large amount and diversity of microorganisms to the soil^56–58^. On the other hand, N in compost is mainly in the organic form, principally as proteins^59,60^. In our experiment, compost amendments supplied large amounts of dissolved organic N, BRSP insoluble N, and organic C (Tables 1 and 3). These substrates are appropriate for nourishing microorganisms, but they are not suitable for plant growth in the short-term. In addition, while biochar amendments can improve soil conditions for microbial colonization and growth^26^, their impact on microbial activity has shown inconsistent results. In this study, biochar did not significantly change the FDA hydrolysing activity nor the CO_2_ emitted (Table 4). Considering biochar's inherent resistance to decomposition, its minor effect on soil CO_2_ respiration is not surprising. Meta-analyses have revealed no notable shifts in soil respiration following the addition of biochar^61^. On the other hand, although not many studies have evaluated FDA hydrolysing activity after biochar amendment, positive responses have been found in salt affected soils^62^ or soils polluted with heavy metals^63^. However, its effect on healthy soils has generally been less favourable^64^.

### Impact of the organic amendments on crop yield and orchard nutritional status

Regarding the orchard nutritional status, no significant effects were found for the different treatments, neither in crop yield nor in leaf nutritional status (Table 5). This was expected, as 25 year-old trees have large canopies and large root systems that accumulate large amounts of nutrient reserves, both as minerals and as carbohydrates. In a related context, Fernández-Escobar et al.^48^, who studied the effect of differential N fertilization on adult (12 and 50 years old) olive trees over 13 years, did not find any effects neither on tree growth nor in crop yield. To identify an effect on soil fertility that could be hidden in adult plants, a bioassay was carried out with young plantlets grown in pots. In this bioassay, biochar alone and especially when blended with compost, resulted in the most favourable outcomes, whilst compost alone did not differ significantly from the unamended soil (Table 6). These results could be only minimally dependent on the mineral nutrition supplied by the amendments, as the olive plantlets in the pot experiment were supplied with a slow release fertilizer to sustain their growth, which minimizes the nutrient contribution of the organic amendments. Similar results were reported by D’Hose et al.^16^ when growing different crops in soils amended with compost, biochar, or their combination, in addition to mineral N and P fertilizer. In our bioassay, the analysis of the leaves (Table 6) indicated that all plants had an adequate content of the main nutrients, with the exception of Ca, which was found to be deficient^65^. Comparatively, plants growing in the compost-biochar-blend amended soil assimilated more N, P, and K than the other plants. Although N and P concentrations were similar in the leaves of these plants than in those grown in the unamended soil, the former accumulated more biomass, and hence, contained a larger total amount of these elements. Potassium was supplied in large amounts by compost (Table1), whilst biochar, contrary to the results of Glaser et al.^32^, reduced the amount of water-soluble K at the same time that it increased that of Mg in soil. This difference could be due to the different pH of the soil used by Glaser et al.^32^, which was slightly acidic (6.3), while ours was markedly alkaline (8.6). The availability of Ca and Mg was lower in the compost-amended soils than in the others (Table 3). This could also be related with soil pH, as in alkaline conditions, Ca and Mg are poorly soluble^66^. Compost increased the per se high soil pH, from 8.6 in the unamended soil to 9.1 in the compost plus biochar amended treatment (Table 3).

Regarding soil fertility, we must consider the potential role of root mycorrhization, as it favours the intake of nutrients by olive trees^67,68^. The case of phosphorous is especially relevant due to its low solubility in alkaline soils^64^. Previous studies indicated that mycorrhization of olive plants had a limited role in increasing P intake and plant growth in soil with low P availability^68,69^, whilst in soil with high P availability, P intake and plant growth was not affected at all^68^. The Bradford-reactive soil protein (BRSP) is accepted as an indirect indication of the content of glomalin in soils, which is a member of the glyco-protein family secreted by mycorrhizal fungi^44,70^, which helps in the production of aggregates in soil^70^. Our results showed that compost alone or mixed with biochar increased the content of BRSP in soil (Table 3), suggesting a stimulatory effect of root mycorrhization. Nevertheless, this hypothesis contrasts with the poor effect of compost on plant growth and plant nutrient acquisition (Table 6). This discrepancy can be explained by the fact that the Bradford assay is not specific for glomalin but for all proteins^44,70^, and by the fact that the extraction procedure followed in or study extracted not only glomalin, but also protein from a wide range of organic sources^71^. Biochar, meanwhile, did not affect the amount of BRSP in our study. This is in accordance with the results by Changxun et al.^72^, who found that biochar did not affect the colonization of citrus roots by arbuscular mycorrhizal fungus (AMF). Nevertheless, these authors found that biochar changed and improved the AMF species composition in soil, which was not evaluated in our study, but may partly explain the stimulating effect of biochar on plant growth.

### Benefits of combining compost and biochar

As observed in this study, compost and biochar can improve soil characteristics and plant growth conditions through different mechanisms. This leads to the hypothesis that a combination of compost and biochar may provide a synergistic effect, and several studies support this hypothesis. Liu et al.^33^ demonstrated a positive synergistic effect of compost and biochar mixtures on soil organic-matter content, nutrient levels, and water-storage capacity of a sandy soil under field conditions. Glaser et al.^32^ found that a blend of compost with biochar increased maize yield by 26% as compared to pure compost. Khorram et al.^53^ reported better results on soil quality when a blend of compost with biochar was applied to a replanted apple orchard than when only compost was applied. On the contrary, D’Hose et al.^16^ did not find any improvement of biochar over the benefits from compost alone on soil and crops.

In the present study, regarding the compost-biochar blend, the results were similar to the compost or biochar alone or even slightly improved (i.e. root growth; number of leaves; N and P soil content; microbial enzymatic activity).

### Economic considerations

In the circumstances of this study, the compost was prepared on-site, as indicated by Sanchez-García et al.^10^, taking advantage of the organic wastes generated in the farm (i.e. two-phase olive mill waste, sheep manure and olive tree pruning). Hence, only the biochar production cost must be considered. The biochar, for his part, was produced and provided free of charge by Proininso Inc. (Málaga, Spain) within the EU project FERTIPLUS (www.fertiplus.eu).

In the case of farmers unable to produce their own compost and biochar, it is pertinent to carry out a basic economic analysis. Compost is a relatively chip material, in the range of 30 to 70 € t^−1^^9,73^. Biochar, on the contrary, is an expensive material which price has been reported to vary from 400 to 1000 £ t^−1^ in UK^74^ and from 300 to 2000 € t^−1^ in the European markets^75^, being in Spain about 400 € t^−1^. In our study, we have applied on average 3 t ha^−1^ year^−1^ of the organic amendments. This represents a mean cost of 150€ ha^−1^in the case of the compost, 1200€ ha^−1^ in the case of biochar, and 255€ ha^−1^ in the case of the mix of 90% compost/10% biochar. Considering that the mean profit of an olive orchard in Spain is 12,000€ ha^−1^ year^−1^^76^, the application of biochar will be unaffordable for farmers. Nevertheless, the mix of compost/biochar at a ratio 90:10 (% dw:dw), as proposed in this study, could probably be profitable for farmers in the medium term.

## Conclusions

The results of this 5-year study indicated that the biannual application of organic amendments generally had a positive impact on the physical properties and the fertility of the soil. Biochar mainly improved the soil physical properties, while compost improved the microbial activity and the nutritional status of the soil, especially regarding the levels of available N (DON and NO_3_^-^). Although it was not possible to detect any effects on adult plants in the orchard, the plantlet bioassay showed a remarkable beneficial effect caused by the mix of biochar and compost, even larger than that caused by biochar or compost separately. This could be due to a synergic effect in the blend that amplifies the good properties of both materials.

Based on these results, and considering some additional facts, such as the desirable long-residence time of biochar in soil, and the low price and high availability of compost in comparison with biochar, we propose the blend tested in this study (90% compost:10% biochar) as an effective starting point for creating high-quality organic soil amendments.

## Data Availability

The datasets used and/or analysed during the current study available from the corresponding author on reasonable request.
